# Development of a recombinase-aided amplification assay for rapid detection of human norovirus GII.4

**DOI:** 10.1186/s12879-021-05942-x

**Published:** 2021-03-09

**Authors:** Zhiwei Qin, Liang Xue, Weicheng Cai, Junshan Gao, Yueting Jiang, Jiale Yang, Yanhui Liang, Linping Wang, Jumei Zhang, Yongdan Hu, Qingping Wu

**Affiliations:** 1 Guangdong Provincial Key Laboratory of Microbial Culture Collection and Application, State Key Laboratory of Applied Microbiology Southern China, Institute of Microbiology, Guangdong Academy of Sciences, No. 100, Xianlie Zhong Road, Guangzhou, Guangdong 510070 People’s Republic of China; 2 Faculty Agriculture and Food, Kunming University of Science and Technology, Kunming, 650500 Yunnan China; 3 Department of Laboratory Medicine, First Affiliated Hospital of Guangzhou Medical University, Guangzhou, Guangdong 510120 People’s Republic of China

**Keywords:** Norovirus, Detection, Rapid diagnostic technique, Recombinase-aided amplification

## Abstract

**Background:**

Human noroviruses are one of the main causes of foodborne illnesses and represent a serious public health concern. Rapid and sensitive assays for human norovirus detection are undoubtedly necessary for clinical diagnosis, especially in regions without more sophisticated equipment.

**Method:**

The rapid reverse transcription recombinase-aided amplification (RT-RAA) is a fast, robust and isothermal nucleic acid detection method based on enzyme reaction. This method can complete the sample detection at 39 °C in 30 min. In this study, we successfully established a rapid reverse transcription recombinase-aided amplification (RT-RAA) assay for the detection of human norovirus GII.4 and applied this assay to clinical samples, as well as comparison with commercial reverse transcription real-time fluorescence quantitative PCR (RT-qPCR).

**Results:**

At 95% probability, the detection sensitivity of RT-RAA was 3.425 log10 genomic copies (LGC)/reaction. Moreover, no cross-reaction was observed with other norovirus genogroups and other common foodborne viruses. Stool samples were examined by RT-RAA and reverse transcription quantitative real-time polymerase chain reaction (RT-qPCR). Compared of RT-qPCR, kappa values for human norovirus detection with RT-RAA were 0.894 (*p* < 0.001), indicating that both assays were in agreement.

**Conclusion:**

This RT-RAA assay provides a rapid, specific, and sensitive assay for human norovirus detection and is suitable for clinical testing.

## Background

Noroviruses (NoV) are positive-sense single-stranded RNA viruses and are regarded as one of the main causes of acute gastroenteritis worldwide. Globally, it is estimated that the disease incidence of NoV has reached approximately 10% and results in over $60 billion in social costs per year [1]. Infection with NoV generally results in abdominal pain, diarrhea, and vomiting [2]. It been noted that NoV infects people of all ages, but is more severe in children and the elderly, especially in low-income countries, leading to over 200,000 deaths every year [3].

Traditional detection methods for NoV, such as immunoassays and electron microscopy, are not suitable for rapid diagnosis because most of them are generally time-consuming and laborious. Common nucleic molecular diagnostic assays for NoV detection with high specificity and sensitivity, such as fluorescence-based real-time PCR and nested PCR, have been used in disease diagnosis [4, 5]. However, expensive equipment and the need for trained technicians make these traditional detection methods restrictive and impractical for rapid and convenient detection in low-income regions.

The recombinase-aided amplification (RAA) assay is a new isothermal nucleic acid amplification technology in recent years for pathogen detection. The reaction is typically completed in approximately 30 min at 37–42 °C. RAA has been successfully applied in the detection of pathogens [6–10] and single nucleotide polymorphisms (SNPs) [11]. Owing to its speed, low-cost, and high sensitivity, RAA is highly suitable for clinical applications, and is a potential assay for the point-of-care testing (POCT) for foodborne pathogens.

In the past two decades, NoV GII.4 has been reported to be the predominant genotype worldwide, and more than half of the outbreaks and sporadic infections are caused by this genotype [12]. For decades, the most popular method for specific detection of NoV GII.4 has been sequencing and phylogenetic analysis [13–15]. Currently, there is no rapid detection method for NoV GII.4. Therefore, it is important to establish an efficient and rapid detection method in response to outbreaks of NoV GII.4. In this work, we describe a novel, isothermal, reverse transcription recombinase-aided amplification (RT-RAA) method for the detection of NoV GII.4.

## Methods

### Virus stock and clinical samples

Positive stool samples of norovirus, enterovirus, astrovirus, adenovirus, sapovirus, and rotavirus used in this study were collected from our previous studies [16]. All samples were diluted to 20% (w/v) in phosphate-buffered saline (PBS, pH 7.2), divided into 50 μL per tube, and stored at − 80 °C until use.

### Nucleic acid extraction

Total RNA was extracted from 50 μL of each stool sample using the High Pure viral RNA kit (Magen, Guangzhou, China) according to the manufacturer’s protocol. The RNA was eluted in 50 μL RNase-free water and stored at − 80 °C until use.

### Design of primers and probes

Complete genome sequences of human norovirus GII.4 strains were obtained from GenBank and used for comparative analyses using MEGA version 7.0 [17]. As the specific site of NoV GII.4 exhibited substantial divergence from other prevalent NoVs (GII.2, GII.3, GII.6, GII.8, and GII.17) and other common foodborne viruses (enterovirus, astrovirus, adenovirus, sapovirus, and rotavirus), it was used as the nucleic target for the RT-RAA of NoV GII.4. The forward and reverse primers and probe were designed according to the manufacturer’s guidelines (Qitian, JiangSu, China) (Table 1). All primers and probes were synthesized by Shanghai GENEray (Shanghai, China).

**Table 1:** The matching of primers and probes

Primer Name	Sequence (5′ → 3′)	Nucleotide	Length	Matching of primer and probe		
				0–2 bp	3–4 bp	≥5 bp
RF1	ATTTTTACGTGCCCAGACAAGAGCCAATGTTCAG	4986–5019/	34 bp	96.4%(53/55)	0 (0/55)	3.6%(2/55)
RF2	CAAGAGCCAATGTTCAGATGGATGAGATTCTCAG	5003–5036/	34 bp	96.4%(53/55)	0 (0/55)	3.6%(2/55)
RR1	TCAGATGGGTTGGCGTCACTCGACGCCATCTTC	5087–5119/	33 bp	96.4%(53/55)	3.6%(2/55)	0 (0/55)
RR2	GACCCATCAGATGGGTTGGCGTCACTCGACGCC	5093–5125/	33 bp	92.7%(51/55)	7.3%(4/55)	0 (0/55)
RR3	TTGGCTGTGGACCCATCAGATGGGTTGGCGTC	5103–5134/	32 bp	96.4%(53/55)	3.6%(2/55)	0 (0/55)
RR4	ACGAGGTTGGCTGTGGACCCATCAGATGGGTTGGC	5106–5140/	35 bp	96.4%(53/55)	3.6%(2/55)	0 (0/55)
RR5	ACCAGGGGCTTGTACAAAATTGTTTCTAATCCAG	5234–5267/	34 bp	94.5%(52/55)	5.5%(3/55)	0 (0/55)
RR6	TTCTAGGGGATACTGTAAACTCTCCACCAG	5263–5292/	30 bp	100%(55/55)	0 (0/55)	0 (0/55)
RR7	TGTTTCTAATCCAGGGGTCAATTACATTTTGT	5216–5247/	32 bp	98.2%(54/55)	1.8%(1/55)	0 (0/55)
RR8	AGCCATAACCTCATTGTTGACCTCTGGGACGAG	5136–5168/	33 bp	98.2%(54/55)	1.8%(1/55)	0 (0/55)
RR9	TGGCCAAATGGGAAAGGTAGGGGTTCAGATCAG	5332–5364/	33 bp	49.1%(27/55)	45.4%(25/55)	5.5%(3/55)
RR10	ATTCTGGCCAAATGAGAAAGGTAGGGATTCAG	5337–5368/	32 bp	47.3%(26/55)	47.3%(26/55)	5.5%(3/55)
RR11	TATTTCACCTGGAGCGTTTCTAGGGGATACTG	5278–5309/	32 bp	96.4%(53/55)	3.6%(2/55)	0 (0/55)
ROPa	TCAGACCTGAGCACGTGGGAGGGCGATCGCAAFHQGGCTCCCAGTTTTGT	5033–5082/	50 bp	96.4%(53/55)	3.6%(2/55)	0 (0/55)

^a^For probe modifications: F = dT-FAM; H = THF; Q = dT-BHQ1. The probe has a 3 C3-spacer for blocking extension

### Preparation of viral standards

The primers F3 and G2SKR were as described in previous studies [18, 19]. A 413-bp (net 4977–5389, GenBank accession no. JX989074) fragment of the ORF1-ORF2 genes of NoV GII.4 was cloned into the pEASY-T1 Vector (Transgen Biotech, Beijing, China) for DNA copy number quantification. Then, the recombinant plasmid was amplified using the Premix Ex Taq Version 2.0 Kit (Takara, Dalian, China) and 2 μL template. The nucleotide amplicon was gel purified using the Hipure Gel Pure DNA Mini Kit (Magen) and subjected to in vitro transcription and recovery of RNA using the mMESSAGE mMACHINE™ T7 Transcription Kit (Thermo Fisher, Shanghai, China) depending on the manufacturer’s instructions. The cRNA was quantified using an EPOCH2 Microplate Spectrophotometer (BioTek, Winooski, VT) and the cRNA copy number was calculated using the following formula: cRNA copy number (copy number/μL) = [6.02 × 10^23^ × cRNA concentration (ng/μL) × 10^− 9^]/[cRNA length in nucleotides × 340]. The cRNA was aliquoted into centrifuge tubes every 5 μL/tube, and stored at − 20 °C until further use.

### RT-qPCR assay

RT-qPCR reactions were carried out using the One Step PrimeScript™ RT-PCR kit (Perfect Real Time, Takara). Each 20-μL reaction mixture contained 4 pmol of forward and reverse primers, 8 pmol of probe, 10 μL of 2× One Step RT-PCR buffer, 2 μL of template, 0.4 μL of TaKaRa Ex Taq HS, 0.4 μL of PrimeScript RT Enzyme Mix II, and RNase Free dH_2_O added to make the volume 20 μL. The primer pairs were COG2R and QNIF2d [20]. The size of the amplification product was 89 bp. The following amplification conditions were used: 42 °C for 300 s, 95 °C for 10 s, followed by 45 cycles of 95 °C for 5 s, and 60 °C for 20 s.

### RT-RAA assay

RT-RAA reactions were carried out using the modified RAA Kit (Qitian). Each 50-μL reaction mixture contained 21 pmol of forward and reverse primers, 6 pmol of probe, 25 μL of buffer VI, 2.5 μL of template, 1 μL of RNase inhibitor (20 U), 1 μL of RTase (50 U) and nuclease-free water added to make the volume 47.5 μL. The reaction mixture was added to RT-RAA lyophilized enzyme pellets, and 2.5 μL of magnesium acetate was added to the tops of the reaction tube lids. The magnesium acetate droplets were then spun down using a mini centrifuge, and the reactions were quickly transferred to a Lightcycler@96 (Roche, Basel, Switzerland) set to 39 °C with cycle reads every 30 s. Nuclease-free water was used as a negative control in every test.

### Evaluation of specificity and sensitivity

Enterovirus, astrovirus, adenovirus, sapovirus, and rotavirus were used to evaluate specificity. NoV GII.2, NoV GII.3, NoV GII.6, NoV GII.8, and NoV GII.17 were used to evaluate the specificity of NoV genotypes. The sensitivity of the RT-RAA assay for detection of human NoV GII.4 was identified using 10-fold dilutions of cRNA (10^7^ to 10^0^ copies per reaction, *n* = 8). Negative control reactions were conducted in parallel for each run.

### Evaluation of the RT-RAA assay using clinical samples

To evaluate the clinical performance of RT-RAA, a total of 38 clinical samples previously confirmed as NoV GII.4-positive by sequencing assay were used for nucleic acid extraction, and detection in the RT-RAA and RT-qPCR assays.

### Statistical analysis

To determine the detection limit of RT-RAA, a probit analysis was performed at a probability level of 95%, and the *p* and kappa values of RT-RAA and RT-qPCR were calculated. All statistics and analysis were performed by using SPSS 24.0 (IBM Corp, Armonk, NY).

## Results

### Development and screening of RT-RAA primer and probe sets

**Fig. 1:**
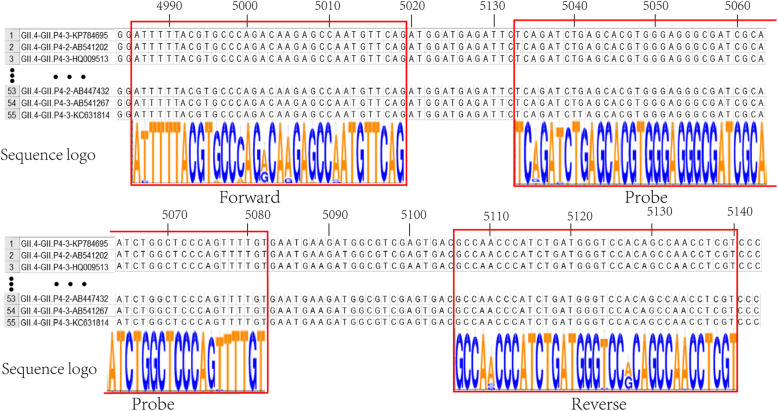
Multiple sequence alignment of primers and probe sets with GII.4 strain sequences

A total of 1942 complete genome sequences of NoVs GII were retrieved from GenBank. Based on bioinformatic analysis, including length, genotype, regional distribution, and prevalence, the non-human NoV strains, repeated sequence strains, obvious long fragment insertion strains, deletion mutations, and long genetic distance strains were removed to obtain 216 reference strains that contained 55 GII.4, 5 GII.1, 20 GII.2, 20 GII.3, 7 GII.5 20 GII.6, 8 GII.7, 1 GII.8, 2 GII.10, 20 GII.12, 6 GII.13, 5 GII.14, 1 GII.16, 20 GII.17, 1 GII.20, 2 GII.21, 3 GII.22, 15 GII.24, and 5 GII.25. Then, the 55 NoVs GII.4 were used as reference strains for comparative analyses to design primers and probes. Twenty-two combinations of candidate primers (2 forward and 11 reverse) were produced and screened for reactivity to purified GII.4 RNA. Of these, five primer sets were identified as capable of amplifying target RNA, and a probe (ROP) was designed to accommodate all sets (Table 1). All RT-RAA primers were screened by fluorescence RAA and electrophoresis with PCR and RAA. Because of resource constraints, one set of primers (RF1 and RR4 with probe ROP) was chosen for subsequent evaluation. Fifty-five GII.4 sequences were used for alignment with primers RF1, RR4, and probe ROP, and sequence alignment showed that RF1RR4 with ROP was highly conserved for GII.4 (Fig. 1).

### Specificity of RT-RAA

**Fig. 2:**
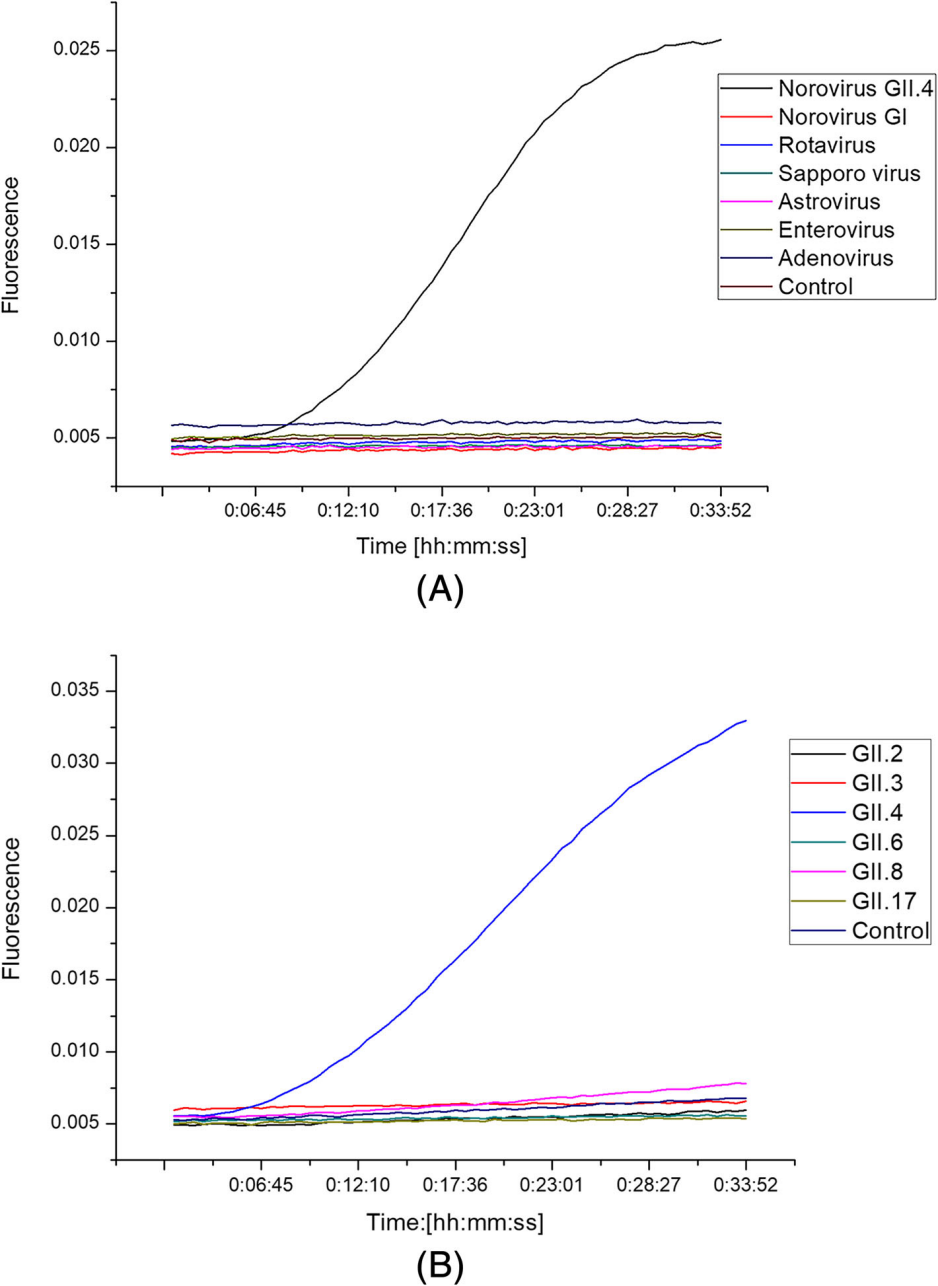
Specificity of RT-RAA for NoV GII.4. **a** The RT-RAA assay was positive for NoV GII.4 and negative for NoV GI, rotavirus, sapovirus, astrovirus, enterovirus, adenovirus, and the negative control. **b** The RT-RAA assay was positive for NoV GII.4, and negative for NoV GII.2, GII.3, GII.6, GII.8, GII.17, and the negative control

The RT-RAA assay was positive for NoV GII.4 and negative for NoV GI, rotavirus, sapovirus, astrovirus, enterovirus, adenovirus, and the negative control (Fig. 2a). The RT-RAA assay was positive for NoV GII.4, and negative for NoV GII.2, GII.3, GII.6, GII.8, GII.17, and the negative control (Fig. 2b). No cross-reactivity of RNA from any control virus was observed. Therefore, RT-RAA for the detection of NoV GII.4 demonstrates a high specificity for the target.

### Sensitivity of RT-RAA

**Fig. 3:**
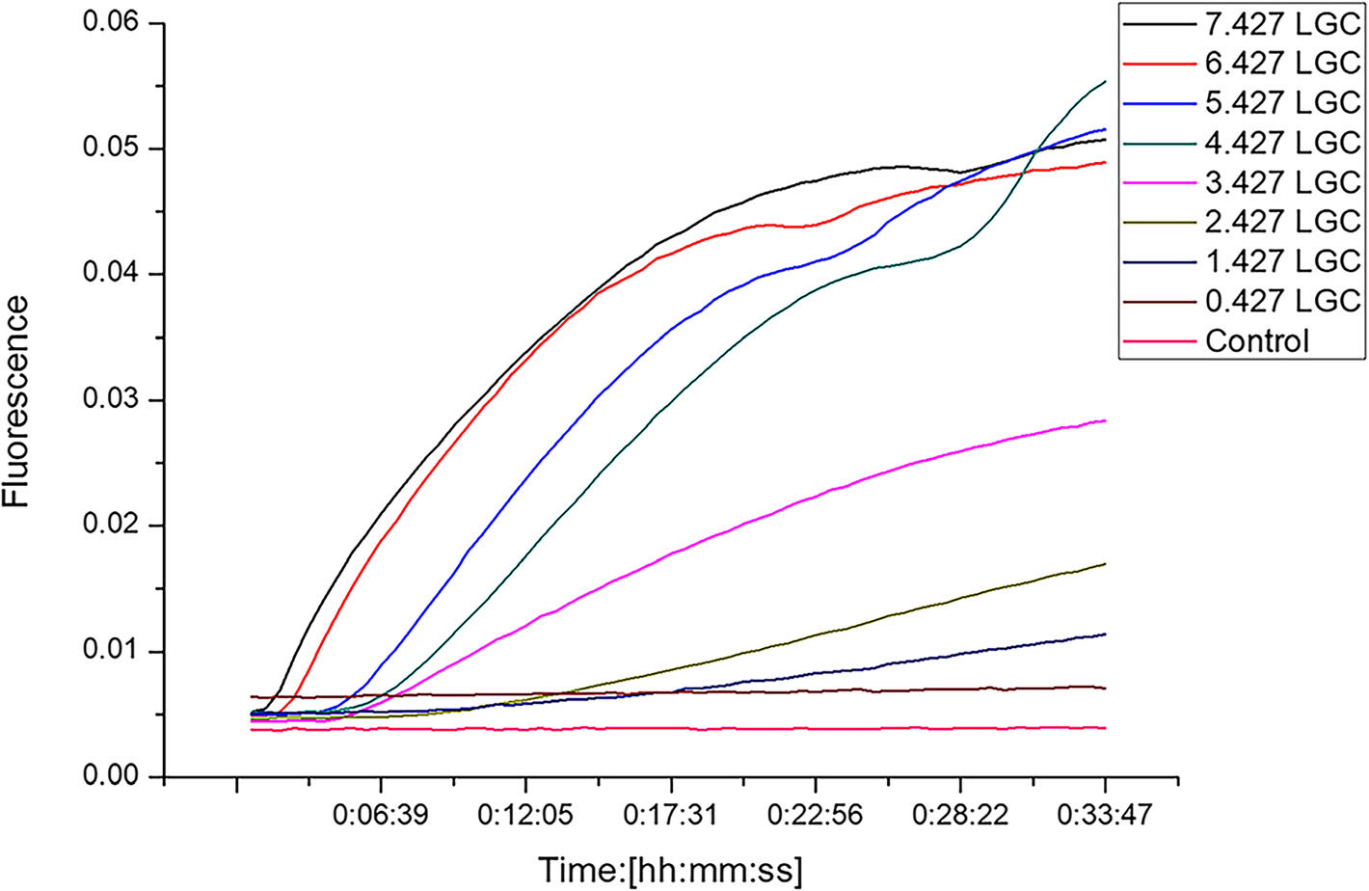
Sensitivity of RT-RAA for GII NoV

After serial dilution from 2.67 × 10^7^ to 2.67 × 10^0^ copies/μL, the cRNA of NoV GII.4 was tested using RT-RAA. The results showed that the detection limit of the RT-RAA was 3.425 LGC (95% Cl: 2.906 LGC–4.471 LGC)/reaction, and the 95% detection limit of the qRT-PCR was 2.110 LGC (95% Cl: 1.586 LGC–3.113 LGC)/reaction (Fig. 3; Table 2).

**Table 2:** Detection limits of human NoVs GII.4 in RT-qPCR and RT-RAA assays

Copies/reaction	Times of positive sample tested by two different assays for NoV (*n* = 8)	
	RT-qPCR	RT-RAA
2.67 × 10^7^	8	8
2.67 × 10^6^	8	8
2.67 × 10^5^	8	8
2.67 × 10^4^	8	8
2.67 × 10^3^	8	7
2.67 × 10^2^	8	6
2.67 × 10^1^	6	1
2.67 × 10^0^	2	0

### Comparison of the RT-RAA and RT-qPCR assays with clinical samples

A total of 38 clinical samples were used to evaluate the RT-RAA assay and RT-qPCR. The RT-qPCR showed positive results for 18 out of 38 samples with a positive rate of 47.4%, and RT-RAA showed positive results for 16 out of 38 samples with a positive rate of 42.1%. Compared with RT-qPCR, the kappa value of the RT-RAA assay for NoV GII.4 detection was 0.894 (p < 0.001), suggesting that both assays were consistent. A detailed comparison of both assays is shown in Table 3.

**Table 3:** Clinical performance of RT-RAA for detection of NoV GII.4

RT-qPCR	RT-RAA		Total	Agreement	Kappa	*p*-value of kappa
	Positive	Negative				
Positive	16	2	18	88.9%	0.894	< 0.001
Negative	0	20	20			
Total	16	22	38			

## Discussion

In recent years, norovirus outbreaks have gradually increased worldwide. For example, it is estimated that there are 56,000–71,000 hospitalizations and 570–800 deaths due to NoV infection every year in the United States [21]. The development of diagnostic technology for this virus is of far-reaching practical significance to improve the quality of public health safety. In this study, we set up a rapid, sensitive, and specific RT-RAA assay for the detection of human NoV GII.4.

Established methods for detecting norovirus, such as conventional RT-PCR [22], droplet digital PCR [23], TaqMan-based real-time RT-qPCR [24], recombinase polymerase amplification (RPA) assays [25], and loop-mediated isothermal amplification (LAMP) assays [26], have multiple limitations. Conventional PCR has limitation of relatively low sensitivity, and requires agarose gel electrophoresis as the final interpretation of the results. TaqMan-based real-time RT-PCR requires expensive thermal cycler devices and trained technicians. The assays based on RPA are more expensive than other detection methods, costing approximately $15 per reaction. LAMP assays are more sophisticated because at least two pairs of primers need to be designed. Therefore, the development of a fast, cheap, and effective assay to detect norovirus is desirable.

The sensitivity of the newly established RT-RAA assay reached 3.245 LGC per reaction. The kappa values of RT-RAA and RT-qPCR indicated that both assays were highly consistent. The few inconsistencies observed might stem from different principles for the two methods and the complexity of clinical samples. Notably, the RT-RAA assay was so rapid that produced positive signal approximately 5 mins, and the entire detection protocol could be completed within 30 mins. In contrast, the RT-qPCR and RT-LAMP assays normally require 1.5 h for completion [27, 28]. Finally, due to thermal cycler limitations, PCR-based detection assays were difficult to integrate in small-scale portable devices amenable for POCT. Compared with LAMP, RT-RAA also effectively avoids the problem of aerosol pollution because the entire assay is completed in the reaction unit without additional opening of the lid. Thus, RT-RAA might be a potential method for POCT of norovirus.

Although the RT-RAA assay has many advantages for detecting foodborne viruses, it also has some limitations. Firstly, it is more difficult to achieve multiplex detection of pathogens using RT-RAA than assays based on PCR because the long primers and probes form dimers more easily [29]. Furthermore, compared with digital PCR, the RT-RAA assay cannot achieve quantitative detection of nucleic acids at current stage. In the future, we will combine the RT-RAA assay with microfluidic digital chip technology for quantitative detection without the need for a thermal cycler or extensive technical expertise, which is expected to become one of the most powerful tools for the rapid and accurate diagnosis of pathogens.

## Conclusions

In conclusion, we successfully established a valuable and alternative RT-RAA analysis for NoV GII.4 detection with low cost, high sensitivity and short time consumption. This new assay can be a suitable method for the diagnosis of NoV infection in poorly developed areas under restricted conditions.

## Data Availability

The datasets used and/or analysed during the current study are available from the corresponding author on reasonable request.

## Abbreviations

RT-RAA: Reverse transcription recombinase-aided amplification
NoV: Norovirus
LGC: Log10 genomic copies
SNPs: Single nucleotide polymorphisms
POCT: Point-of-care testing
RPA: Recombinase polymerase amplification
LAMP: Loop-mediated isothermal amplification
RT-qPCR: Reverse transcription real-time fluorescent quantitative PCR
RT-PCR: Reverse transcription PCR

## References

[1] Bartsch SM, Lopman BA (2016). Global economic burden of norovirus gastroenteritis. PLoS One.

[2] Moore MD, Goulter RM (2015). Human norovirus as a foodborne pathogen: challenges and developments. Annu Rev Food Sci Technol.

[3] Bányai K, Estes MK (2018). Viral gastroenteritis. Lancet.

[4] Yan H, Yagyu F (2003). Detection of norovirus (GI, GII), sapovirus and astrovirus in fecal samples using reverse transcription single-round multiplex PCR. J Virol Methods.

[5] Yoo JE, Lee C (2017). Evaluation of various real-time reverse transcription quantitative PCR assays for norovirus detection. J Microbiol Biotechnol.

[6] Bai X, Ma X, et al. Field applicable detection of hepatitis B virus using internal controlled duplex recombinase-aided amplification assay and lateral flow dipstick assay. J Med Virol. 2020; 10.1002/jmv.25778. 10.1002/jmv.25778 32190907

[7] Li Y, Yu Z (2020). Development of a recombinase-aided amplification assay for rapid and sensitive detection of porcine circovirus 3. J Virol Methods.

[8] Wang Y, Cui Y (2020). Development of a recombinase-aided amplification assay for detection of orf virus. J Virol Methods.

[9] Xue G, Li S (2020). Reverse-transcription recombinase-aided amplification assay for rapid detection of the 2019 novel coronavirus (SARS-CoV-2). Anal Chem.

[10] Xue G, Li S, et al. Use of a rapid recombinase-aided amplification assay for mycoplasma pneumoniae detection. BMC Infect Dis. 2020b;20(1) 10.1186/s12879-019-4750-4. 10.1186/s12879-019-4750-4 PMC6988361 31992210

[11] Duan S, Li G (2018). A probe directed recombinase amplification assay for detection of MTHFR A1298C polymorphism associated with congenital heart disease. BioTechniques.

[12] Siebenga JJ, Vennema H (2009). Norovirus illness is a global problem: emergence and spread of norovirus GII.4 variants, 2001–2007. J Infect Dis.

[13] Allen DJ, Trainor E (2016). Early detection of epidemic GII-4 norovirus strains in UK and Malawi: role of surveillance of sporadic acute gastroenteritis in anticipating global epidemics. PLoS One.

[14] Nenonen NP, Hannoun C (2014). Norovirus GII.4 detection in environmental samples from patient rooms during nosocomial outbreaks. J Clin Microbiol.

[15] Silva LDD, Rodrigues EL (2013). Detection of the pandemic norovirus variant GII.4 Sydney 2012 in Rio Branco, state of Acre, northern Brazil. Mem Inst Oswaldo Cruz.

[16] Xue L, Dong R (2016). Molecular epidemiology of noroviruses associated with sporadic gastroenteritis in Guangzhou, China, 2013-2015. Arch Virol.

[17] Kumar S, Stecher G, Tamura K. MEGA7: Molecular Evolutionary Genetics Analysis Version 7.0 for Bigger Datasets. Molecular Biology and Evolution. 2016;3(7):1870–4. 10.1093/molbev/msw054. 10.1093/molbev/msw054 PMC8210823 27004904

[18] Kojima S, Kageyama T (2002). Genogroup-specific PCR primers for detection of Norwalk-like viruses. J Virol Methods.

[19] Luo JWXX (2012). Colorimetric detection of norovirus genotype GII by reverse transcription loop-mediated isothermal amplification. Chin J Virol.

[20] Loisy F, Atmar RL (2005). Real-time RT-PCR for norovirus screening in shellfish. J Virol Methods.

[21] Vinjé J (2015). Advances in laboratory methods for detection and typing of norovirus. J Clin Microbiol.

[22] Osazuwa F, Grobler HS, et al. Phylogenetic lineage of GII.17 norovirus identified among children in south-south, Nigeria. BMC Res Notes. 2020;13(1) 10.1186/s13104-020-05185-0. 10.1186/s13104-020-05185-0 PMC7376658 32698856

[23] Persson S, Eriksson R (2018). Comparison between RT droplet digital PCR and RT real-time PCR for quantification of noroviruses in oysters. Int J Food Microbiol.

[24] Gao X, Wang Z (2019). Surveillance of norovirus contamination in commercial fresh/frozen berries from Heilongjiang Province, China, using a TaqMan real-time RT-PCR assay. Food Microbiol.

[25] Moore MD, Jaykus L. Development of a recombinase polymerase amplification assay for detection of epidemic human noroviruses. Sci Rep. 2017;7(1) 10.1038/srep40244. 10.1038/srep40244 PMC5220337 28067278

[26] Yaren O, Bradley KM (2016). A norovirus detection architecture based on isothermal amplification and expanded genetic systems. J Virol Methods.

[27] Jeon SB, Seo DJ (2017). Development of one-step reverse transcription loop-mediated isothermal amplification for norovirus detection in oysters. Food Control.

[28] Zaid Haddadin MDEB. Characteristics of GII.4 norovirus versus other genotypes in sporadic pediatric infections in Davidson County, Tennessee, USA. Clin Infect Dis. 2020; 10.1093/cid/ciaa1001. 10.1093/cid/ciaa1001 PMC8492161 32667045

[29] Yan T, Li X (2018). Development of a reverse transcription recombinase-aided amplification assay for the detection of coxsackievirus A10 and coxsackievirus A6 RNA. Arch Virol.

